# Progress in construction of mouse models to investigate the pathogenesis and immune therapy of human hematological malignancy

**DOI:** 10.3389/fimmu.2023.1195194

**Published:** 2023-08-14

**Authors:** Yue Lang, Yanan Lyu, Yehui Tan, Zheng Hu

**Affiliations:** ^1^ Key Laboratory of Organ Regeneration and Transplantation of Ministry of Education, The First Hospital, Jilin University, Changchun, China; ^2^ Department of Dermatology, The First Hospital, Jilin University, Changchun, China; ^3^ Department of Hematology, The First Hospital, Jilin University, Changchun, China

**Keywords:** humanized mice, PDX model, hematological malignancy, immune therapy, immune system

## Abstract

Hematological malignancy is a disease arisen by complicate reasons that seriously endangers human health. The research on its pathogenesis and therapies depends on the usage of animal models. Conventional animal model cannot faithfully mirror some characteristics of human features due to the evolutionary divergence, whereas the mouse models hosting human hematological malignancy are more and more applied in basic as well as translational investigations in recent years. According to the construction methods, they can be divided into different types (e.g. cell-derived xenograft (CDX) and patient-derived xenograft model (PDX) model) that have diverse characteristics and application values. In addition, a variety of strategies have been developed to improve human hematological malignant cell engraftment and differentiation *in vivo*. Moreover, the humanized mouse model with both functional human immune system and autologous human hematological malignancy provides a unique tool for the evaluation of the efficacy of novel immunotherapeutic drugs/approaches. Herein, we first review the evolution of the mouse model of human hematological malignancy; Then, we analyze the characteristics of different types of models and summarize the ways to improve the models; Finally, the way and value of humanized mouse model of human immune system in the immunotherapy of human hematological malignancy are discussed.

## 1 Introduction

Hematopoietic system is formed by a complex differentiation process (e.g. from hematopoietic stem cells (HSCs) to different lineages of blood/immune subsets) and plays an important role in maintaining oxygen exchange and immune monitoring. Hematopoietic system is controlled by external and internal factors, such as the hematopoietic microenvironment, transcription factors, signal transduction pathways and chromatin modifiers (1). Abnormal changes in any factor may cause serious hematological diseases (2, 3) and endanger human health. Among these diseases, hematological malignancies have the most serious impact on human health.

Hematological malignancy refers to a group of heterogeneous and life-threatening serious diseases mainly caused by abnormal HSCs, which lead to impairment in different stages of the differentiation process, including differentiation block, apoptosis disorder and malignant proliferation (4). According to the American Cancer Society, hematological malignancies account for approximately 6%-10% of all malignancies, and the mortality rate is 5.8% of all malignancies (5 **)**. Therefore, research on the pathogenesis, drug development and therapeutic approaches is of great significance to reduce the incidence and mortality rates of hematological malignancies.

According to the fifth edition of the World Health Organization (WHO) classification of hematolymphoid tumors in 2022 (6 **)**, hematological malignancies mainly include leukemia, lymphoma, myelodysplastic tumors (MDS) and multiple myeloma (MM). Treatment methods for hematological malignancies include chemotherapy, radiotherapy, molecular targeted drug therapy, HSC transplantation, and immunotherapy. Although some types of human hematological malignancies (such as acute promyelocytic leukemia, chronic myelogenous leukemia (CML) and chronic lymphocytic leukemia (CLL)) (7) have been basically eradicated, most types of hematological malignancies still have problems such as low long-term cure rate and high recurrence rate. Moreover, the current treatment methods still require further improvement because of some associated serious side effects (8).

Animal models are an important tool for the study of pathogenesis and drug/therapy of hematological malignancy, and mouse models are most widely used. However, a large amount of evidence shows that the conclusions obtained from conventional mice or drugs/therapies developed for mice cannot be applied to humans. The main reason for this is that there are huge evolutionary differences between rodents and humans (9). To solve this problem, researchers have constructed multiple types of mouse models with human hematological malignancies and applied them to basic research and drug research, which promote the development of this field (10). Recently, immunotherapy has become an important therapy for the treatment of hematological malignancies; The humanized mouse model, which can reproduce the process of human immunotherapy, also plays an increasingly important role in the study of immunotherapy for hematological malignancies.

This review first describes the development of mouse models of human hematological malignancies and their application in preclinical research, summarizes the construction methods and technical characteristics with a comparison of their advantages and disadvantages, and finally discusses future development trends.

## 2 Generation of immunodeficient mouse strains for human hematological malignancy investigation

**Figure 1:**
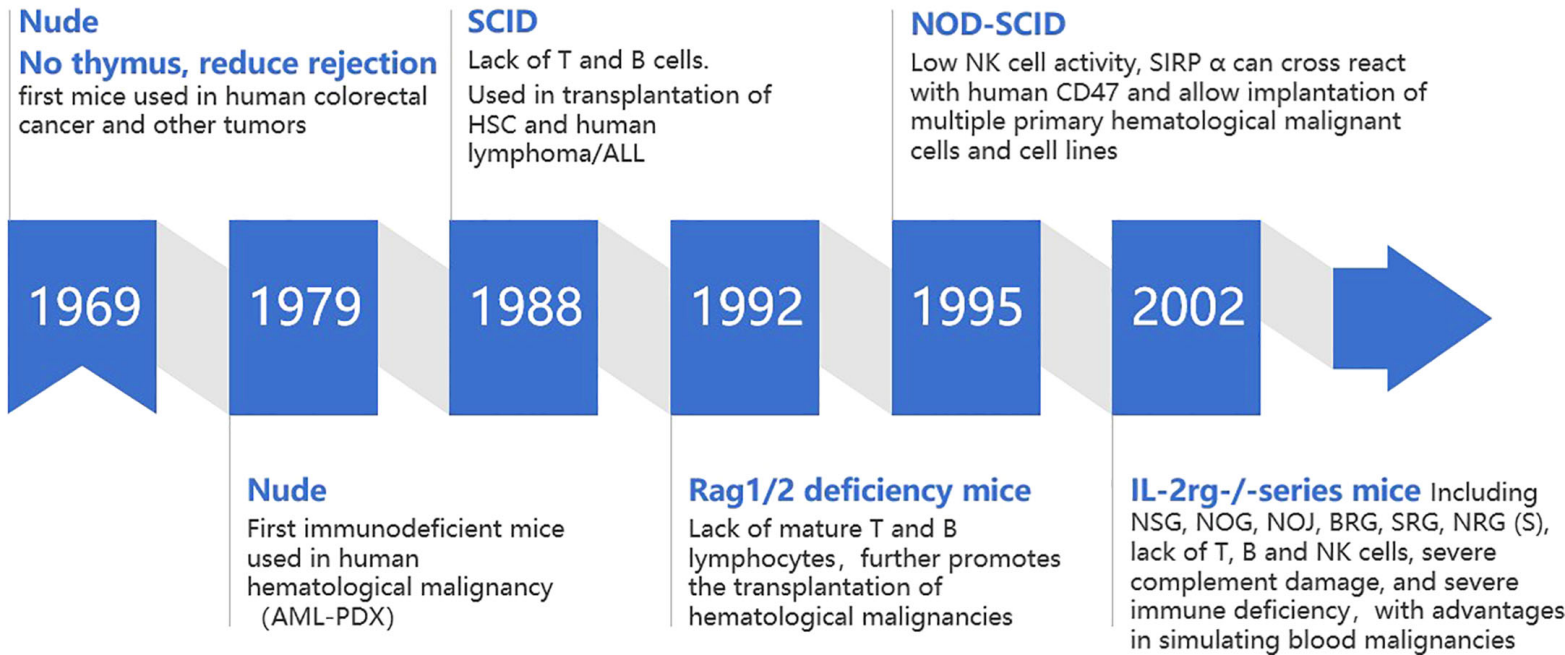
Development of mouse models of human hematological malignancy.

Robust xenogeneic immune reaction is the first obstacle impeding the development of mouse model with human hematological malignant cell repopulation. In this process, both innate and adaptive immune systems play an important role (11). Immunodeficient mice can be prepared by modifying key genes that regulate the development, survival and function of immune cells, which lays the foundation for replicating human hematological malignancy in mice. Therefore, immunodeficient mouse strains play an important role in promoting the development of human hematological malignancy mouse models (**Figure 1**).

### 2.1 Foxn1-/- nude mice

*Foxn1* ^-/-^ nude mice were the first immunodeficient mice used in the study of human malignancies. *Foxn1* ^-/-^ nude mice are knocked out of the forkhead box N1 (*Foxn1*, formerly known as *Why* or *Hfh11*) gene, with congenital absence of thymus and T cells, which can alleviate T-cell-mediated host versus gradient reaction xenograft rejection. Rygaard and Povlsen successfully used nude mice for xenotransplantation of human colon adenocarcinoma for the first time in 1969 and expanded its use to other solid tumors (12 **)**. At the end of 1970s, Franks et al. first tried to establish a subcutaneous implantation model of human acute myeloid leukemia (AML), which was the first time that nude mice were used in the study of human hematological malignancies. However, AML cells began to regress after 6 days in mice (13). The complete innate immune system and B cell humoral immune system of nude mice limit the transplantation rate of tumor cells, especially human hematological malignant cells (14). In general, *Foxn1*-/- nude mice are not suitable to support human hematological malignant cell engraftment because of the residual mouse versus human immunological responses mediated by mouse B cell, natural killer (NK) cell and macrophages.

### 2.2 C.B17-SCID and Rag1/2 deficient mice

In 1988, C.B17-SCID (C.B17-*Prkdc* ^scid^, protein kinase DNA activated catalytic polypeptide) mice were generated. The *prkdc* mutation carried by C.B17-SCID mice prevented the development of T and B cells in the adaptive immune system, resulting in a lack of T and B cells (15). Compared with nude mice, this strain has a higher transplantation rate of human solid tumor cells (16) and was the first immunodeficient mouse that can be used to transplant human HSCs (17) and human lymphoma (18)/acute lymphoblastic leukemia (ALL) cells (19). However, with the increase of age, 2%-23% of functional T and B cells gradually develop in 3-9 months old C.B17-SCID mice. This immune leakiness (20) could inhibit human malignant cell engraftment (20). In addition, the *prkdc* mutation of C.B17-SCID mice can also lead to DNA repair defects, resulting in radiosensitivity (21, 22).

Similar to C.B17-SCID mice, in 1992, Mombaerts (23) and Shinkai (24) produced C57BL/6 background mice with deletion of the recombination-activating gene 1/2 (RAG1/2)- coding region. This defect prevented the recombination of antigen receptor genes and thus made RAG1/2 deficient mice lacking mature T and B lymphocytes. RAG1/2 deficient mice did not exhibit the leakiness and radiosensitivity of C.B17-SCID mice.

However, the intrinsic immune system activity (including high level of NK cell activity) of C.B17-SCID and Rag1/2-deficient mice reduced the implantation rate of human HSCs and tumor cells; NK cells from C.B17-SCID and Rag1/2-deficient mice show cytotoxicity toward human HSCs and tumor-initiating cells (25),further limiting their application in tumor models.

### 2.3 NOD-SCID mice

In 1995, NOD-SCID mice based on NOD background were generated. The NOD-SCID mice with NOD background have inherent immune defects, including decreased NK cell activity and lack of complement C5. The Signal regulatory protein α (*SIRPA*) gene is expressed in myeloid cells and encodes the suppressive immunoglobulin superfamily transmembrane protein CD172a. When combined with CD47, gene acts as a homologous ligand of “don’t eat me” signal to inhibit autoimmune recognition. *SIRPA* of NOD background mice can cross-react with human CD47 (26), resulting in better tolerance of macrophages to human cells. Compared with C.B17-SCID mice, NOD-SCID mice have a higher implantation efficiency of human HSCs and tumor cells of approximately five times. They could implant hematological malignant cells with lower transplantation rate in the past, including cell lines and primary tumor cells such as lymphoma (22) and leukemia (including AML (26),ALL (27)) cells.

### 2.4 *Il2rg*-/- mice

Interleukin-2 receptor γ chain (*Il2rg*) is responsible for the high affinity binding of IL2, IL4, IL7, IL9, IL15, and IL21 (28). Mice with *IL2rg* gene knockout (29), due to the complete lack of NK cells (11), further improved the rate of human tumor cell transplantation.

In 2002-2005,NSG (NOD/LtSz SCID,*IL2rg*-/-)/NOG(NOD/Shi SCID, *IL2rg*-/-) *(* 30)mice were generated, which are NOD-SCID mice with *IL2rg*-/-,not only lacking T,B and NK cells but also complement C5,and showing severe immune deficiency. In addition, the NOD background of NSG/NOG can tolerate macrophage phagocytosis caused by incompatibility of CD47-*SIRPA*, which has advantages in modeling hematological malignancies and solid tumors (31). Except for NSG/NOG, there are other similar immunodeficient mouse models that can be applied to the study of human hematological malignancy *in vivo*, such as NOJ (NOD-SCID/Jak3null) (32),BRG(Balb/c Rag2-/-*IL2rg*-/-) (33),SRG(Transgene(Tg)(human *SIRPA*)Rag2-/-*IL2rg*-/-) *(* 34),NRG(NOD-Rag2-/-*IL2rg*-/-,with radiation resistance (35)),NRGS(cross NRG with NSG-SGM3) (36). Considering their significant advantages, NSG and NOG mice are now widely used to study human hematological malignancy *in vivo* in recent years.

AML, acute myeloid leukemia;PDX, patient derived xenograft;SCID, severe combined immunodeficiency;HSC, hematopoietic stem cell;ALL, acute lymphoblastic leukemia;Rag, recombination-activating gene; NOD, non-obese diabetic; NK, natural killer; *SIRPA*, Signal regulatory proteinα;NSG,NOD/LtSz SCID,*IL2rg*-/-;NOG,NOD/Shi SCID,*IL2rg*-/-;NOJ, NOD-SCID/Jak3null ;BRG, Balb/c Rag2-/-*IL2rg*-/-;SRG, Transgene(Tg) (human *SIRPA*)Rag2-/-*IL2rg*/-;NRG,NOD-Rag2-/-*IL2rg*-/-;NRGS, cross NRG with NSG-SGM3; *IL2rg*, interleukin-2 receptor γ-chain;

## 3 Development of mouse models with human hematological malignancy development

**Figure 2:**
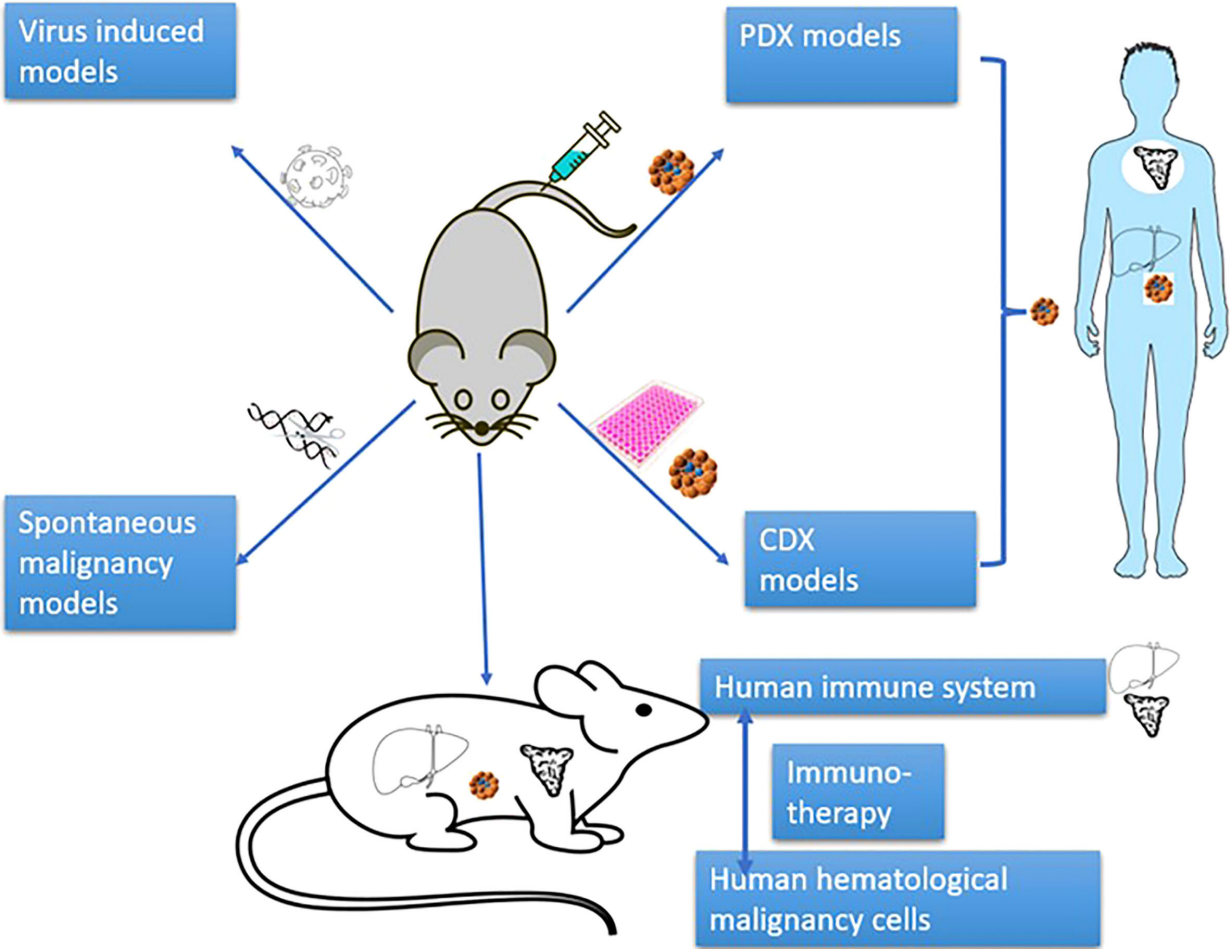
Classification of mouse models of human hematological malignancy.

In terms of the manners to achieve human cell engraftment, human hematological malignancy mouse models mainly include human malignant cell xenotransplantation models (cell-derived xenograft (CDX)) model, patient-derived xenograft model (PDX)), virus-induced models, induced pluripotent stem cell (iPSC)-induced models and spontaneous malignancy models (**Figure 2**). These models have promoted the development of research on the pathogenesis of hematological malignancies and on anti-tumor drugs/therapies.

The xenotransplantation model is defined as the model of transplanting patient cells (PDX) or cell lines (CDX) to immunodeficient mice.

### 3.1 CDX model

In 1990, the National Cancer Institute (NCI-60) tumor cell line became popular in tumor research. This cell line can be cultured *in vitro* with a fast growth rate and easy operation (37). To deeply study the physiology of the human blood-lymphatic system and their respective diseases *in vivo*, the human tumor CDX model was used, which can study the disease mechanism and guide treatment. Since the genotype and phenotype of tumor cell lines are homogeneous and immortal, the CDX model is convenient to study the carcinogenic mechanism, as well as diagnosis and treatment options (38). NOD-SCID or NSG or NOG mice are the strains frequently used to construct human hematological malignancy CDX model. Most CDX models can be constructed by intravenous injection of human cancer cells which may spread over the body as they act in human beings. Whereas MM CDX model requires intra-bone injection to improve cancer cell engraftment rate (39). Compared with PDX model, CDX model is easier to constructed and the stability and successful rate are markedly higher.

However, after transplantation into mice, the tumor cell line lost its original characteristics (40) and could not represent the heterogeneity of clinically complex hematological malignancies (gene expression, growth and invasion characteristics), and the value of predicting clinical prognosis was limited (41, 42).

### 3.2 PDX model

The PDX model of hematological malignancy was established by transplantation of a patient’s primary hematological malignant cells into immunodeficient mice. Unlike the CDX model, the PDX model preserves the original characteristics, tumor heterogeneity, and growth migration and can well represent the disease progression and treatment response of patients (43). Engraftment rates of PDX models may predict the disease outcome in patients. Since PDX models may retain the features of original tumor, harbor close genetic profiles (44), they can be used to identify the chemo-resistant tumor cell population (45, 46).

NSG and NOG mice are frequently used to construct human hematological malignancy PDX models. Human leukemia cells tend to engraft in mouse bone marrow first and then spread over mouse whole body (47). The organ engraftment tendency of other types of human hematological malignant cell may depend on their original characteristics in patients (48–51). According to disease classification, PDX models of hematological malignancies are divided into the following categories:

#### 3.2.1 Lymphoma

Lymphoma is a common malignant proliferative disease of hematopoietic system, which can affect the whole-body organs (52). Traditionally, lymphoma is divided into Hodgkin’s lymphoma (HL) and non-Hodgkin’s lymphoma (NHL), accounting for approximately 10% and 90% of all lymphomas, respectively (53). The first PDX lymphoma model was developed in 1993 (54). In this model, human HL cells were transplanted into unirradiated CB17.SCID mice kidney/liver. The main advantage of the lymphoma PDX model is that it retains the characteristics of primary lymphoma (55), which summarizes the refractory nature of human lymphoma and non-response to treatment (44), to develop new drug targets and personalized drug treatment (56, 57). In terms of inoculation methods, in addition to intravenous injection, implantation into the renal capsule or intraperitoneal cavity has a higher success rate (48). However, it is necessary to pay attention to complications such as bleeding caused by kidney injury (58).

#### 3.2.2 Leukemia

Leukemia is a malignant clonal disease of HSCs (59). Because of uncontrolled proliferation, impaired differentiation, and blocked apoptosis, clonal leukemia cells proliferate and accumulate in hematopoietic tissues, infiltrate other non-hematopoietic tissues and organs, and inhibit hematopoietic function. Similar to the PDX model of lymphoma, the PDX model of leukemia retains its immunophenotype, chromosome aberration, transcriptome and MRD marker expression (60, 61);The onset time of leukemia cells in mice is inversely proportional to the recurrence rate in humans; Moreover, the transplantation of leukemia stem cells (LSCs) can represent the development of leukemia (62, 63), and its efficiency reflects the prognosis of clinical patients (64). Using the LSC PDX model, researchers found genes or subclones related to drug resistance and relapse in patients with cytogenetic remission (65, 66), such as CML (67) and pediatric T cell acute lymphoblastic leukemia (T-ALL) (45). In terms of inoculation method, intravenous implantation can form a leukemia model with systemic spread, which is consistent with the clinical progress of leukemia (68); direct injection into the marrow cavity bypasses the homing process of cells and provides a bone marrow microenvironment suitable for the growth of leukemia cells, with a higher implantation rate (69).

#### 3.2.3 MM

MM is a monoclonal malignant blood tumor characterized by abnormal proliferation of plasma cells in the blood, accompanied by anemia, hypercalcemia, renal failure and osteolytic injury. Owing to its frequent recurrence and drug resistance, the cure rate is extremely low. The MM PDX model can well represent the development process of disease and develop new treatment methods. In the past, mobilized blood mononuclear cells or CD34^+^-enriched cells from advanced MM patients could develop into myeloma in irradiated NOD-SCID mice by intracardiac injection, which allows injected cells to bypass lung and flow directly into bone marrow (70). At present, MM PDX is mainly constructed by injecting primary myeloma cells (MC) intravenously or intra-osseously (39). Intravenous models are easy to operate and can represent the characteristics of “diffused MM syndrome”; while intratibial models are often limited in MC growth and may not model diffused diseases. In addition, MM PDX model constructed by subcutaneous injection manner is utilized to study refractory and recurrent human MM which may own extramedullary effusion feature (71, 72). However, patient-derived MCs in the MM PDX model can grow and colonize in the bone marrow of mice but often do not have bone disease (73). Research on the efficacy of drugs in treating MM-induced bone disease is extremely limited. At this time, the MM CDX model that leads to osteolytic injury is often selected to better study the anti-MM bone disease therapy.

#### 3.2.4 MDS

MDS are a group of heterogeneous diseases of HSCs, characterized by repeated genetic abnormalities (74) resulting in the reduction of the number of hematopoietic cells and morphological dysplasia, which are divided into high-risk MDS and low-risk MDS according to the degree of malignancy. Contrary to AML, research on MDS is hindered by the lack of a preclinical model that replicates the complexity and heterogeneity of the disease (75). The MDS CDX model that summarizes the MDS bone marrow failure status is rare and has limited genetic diversity (76). The MDS PDX model is constructed from MDS stem cells from patients (77), which reproduces human myeloid differentiation rather than lymphoid differentiation (78). By implanting high-risk MDS cells into the PDX model (79), researchers can study whether there are other abnormalities in model-derived bone marrow stem cells (80). However, the success rate of implanting low-risk MDS in NSG mice is still low, which needs to be further resolved (81).

In summary, the PDX model, as a pre-clinical bridge for potential clinical application, is used to guide treatment (82, 83);PDX can simultaneously transplant patient effector cells to evaluate individual efficiency (84) and maintain important characteristics of the original tumor, including histology, genome pattern, cell heterogeneity and drug responsive (85), cell-cell interaction (86, 87), and clonal diversity (88). In addition, through the PDX model constructed by transplanting drug-resistant primary human hematological malignant cells, researchers can determine the drug resistance mechanism or molecular target (43, 89).

### 3.3 iPSC-induced PDX model

The above PDX/CDX models have difficulties in building some hematological malignancy models with low invasion (such as CML), lack of cell lines (such as MDS) or insufficient donor samples. The iPSC-induced PDX model can differentiate patients’ iPSCs into HSPC that regain the ability to cause hematological malignancies *in vivo (* 90). IPSC drug testing system (82) can be used to test the potential treatment methods to reproduce the disease DNA methylation/gene expression pattern and drug resistance (91, 92). In theory, iPSC-induced HSPC can be repeatedly used to build unlimited mouse models based on limited original samples and solve the different copy number changes of PDX models and patients (93, 94). Human CD34^+^ cells were isolated from primary patient bone marrow mononuclear cells cultured in cytokines, and reprogramming factors were transduced to obtain stable iPSC clones. Then, iPSC could be differentiated into HSCs *in vitro* (95) and transplanted into immunodeficient mice to construct the PDX model. Alternatively, iPSCs can be co-injected with OP9 stromal cells into immunodeficient mice where human iPSCs develop into HSCs; and those HSCs can be used for secondary transplantation for PDX model generation (91). At present, patient-derived chronic myelomonocytic leukemia iPSCs, juvenile myelomonocytic leukemia iPSCs (96), AML iPSCs and MDS iPSCs have successfully differentiated into HSCs and PDX models in NSG mice have been built. iPSCs derived from AML patients with MLL rearrangement (97) and MDS-iPSC differentiated HSCs (98) can be used to build PDX models in NSG mice and allow the study of characteristics, drug sensitivity, and prediction of relapse of different subclones. However, CML iPSCs with *BCR-ABL* gene (99) and acute B lymphoblastic leukemia (B-ALL) with MLL rearrangement (100) failed to differentiate into specific HSC, which needs further study. Some teams further glycoengineered HSCs derived from MDS iPSCs and transplanted these cells into NSG mice, obtaining strong progenitor cell activity, bone marrow transport, and exosmosis, but not long-term implantation (101).

### 3.4 Virus induced model

Virus infection often induces hematological malignancies, such as Epstein-Barr virus (EBV)-induced lymphoma, Human T-lymphotropic virus (HTLV) -1-induced ATL (adult T cell leukemia), EBV-induced aggressive NK-cell leukemia. Nearly 50% of HL is induced by EBV (102). The mouse model with human immune system can represent human hematological malignancy induced by a virus interference genome or non-specific activation. Injection of patient HTLV-1 virus infected human PBMCs into NSG mice resulted in T cell clonal proliferation and development of ATL (103); BLT (bone marrow-liver-thymus) humanized mice can also be used to study HTLV-1-induced-ATL (104);The humanized mouse model of lymphoma can be made by infection of EBV in human CD34^+^ HSCs transplanted newly born immunodeficient mice (C.B-17-SCID (105), NOD-SCID (106), NOG (107), NSG (108)). These models reshape the pathogenesis and mechanism of human lymphoma *in vivo*, and replicate the potential infection, T-cell-mediated immune response and humoral immune response *in vivo (* 109, 110). Different conditions (e.g. mouse strain/age, donor cells, viral dosage and so on) may induce lymphoma into different phenotypes. When infected with EBV, the humanized mice with high numbers of human B cells trend to generate, while HL mainly occur in the humanized mice reconstituted with high levels of human T-cells (111). However, the humanized mouse model of lymphoma induced by EBV has differences in the mode of virus transmission. Oral transmission of humanized mice is not feasible because the oropharyngeal epithelial cells of humanized mice are derived from mice. Whereas human lymphoma is mainly transmitted through oral infection, which leads to the heterogeneous immune response of human and mouse. The humanized mouse model of lymphoma made by co-infection of EBV/Kaposi’s sarcoma-associated herpesvirus (KSHV) virus (112) resulted in long term viral infection in humanized NSG mice that representing lymphoma (113) of PEL (primary effusion lymphoma) type. The main manifestations were viral infection in the spleen and abdominal cavity, and the lymphoma showed plasma cell differentiation signs.

### 3.5 Spontaneous malignancy model

**Figure 3:**
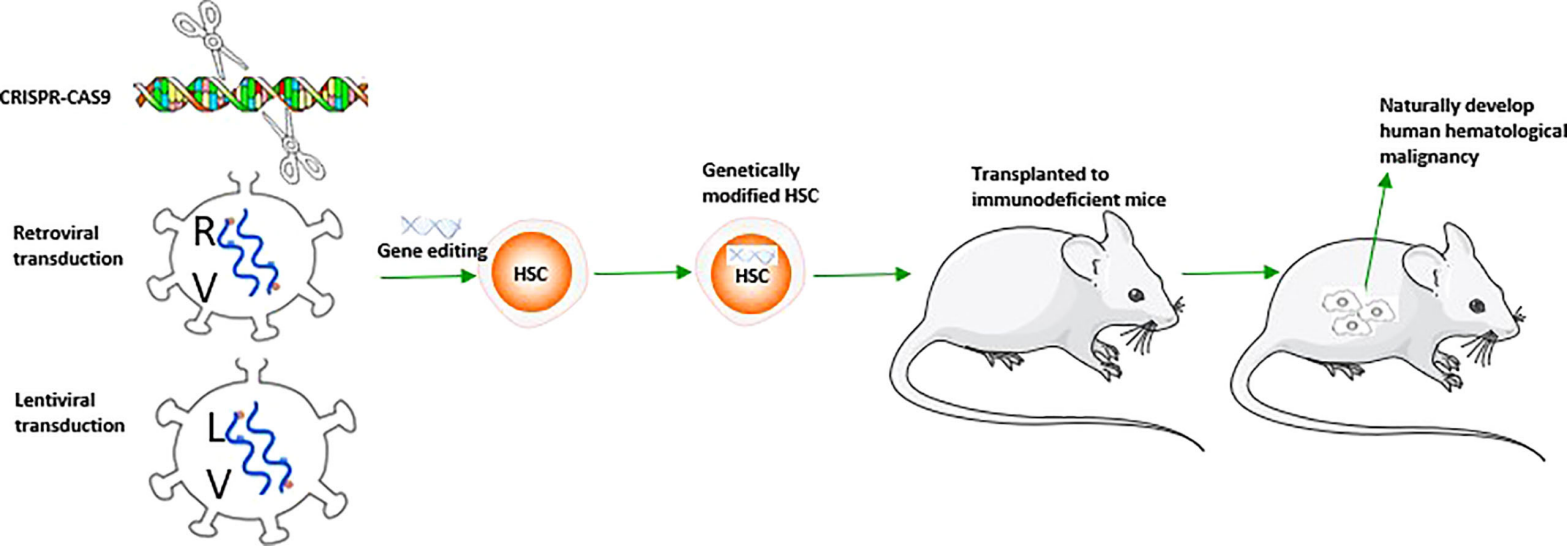
A schematic figure about the construction of mouse model with spontaneous human hematological malignancy development.

In addition to the models mentioned above, the mouse model with human hematological malignancy natural development can be made by genetic engineering modification of the target genes causing hematological malignancy in human HSCs before transplantation into immunodeficient mice. This spontaneous human malignancy mouse model shows similar performance and immune phenotype to patients with the same gene rearrangement, providing a better understanding of the molecular etiology of specific subtypes (**Table 1**). Based on its specific genetic abnormality, it provides the direction for potential treatment. There are many methods of HSC gene editing, including retroviral transduction, lentivirus transduction, and Clustered Regularly Interspaced Short Palindromic Repeats/Cas9 (CRISPR/Cas9) engineering transduction, which have their own characteristics in inducing hematological malignancy (**Table 1**). The solid tumor model requires at least three different carcinogenic genes to transform primary human cells **(** 124, 125 **)**,and the progress is slow. Unlike the solid tumor spontaneous model, the hematological malignancy spontaneous model can quickly obtain the required additional genetic or epigenetic events, and only one or two genes are needed; For example, Lin^-^(lineage negative) and CD34^+^ human UCB (umbilical cord blood) cells transfected by MLL-AF9, MLL-ENL in NOD-SCID (β2M-/-) show pre-B-ALL and AML and mixed pedigree (114, 115);Transfection of CD34^+^ human HSCs with the retroviral vector containing ZYM2-FGFR1 promotes the development of MPD to AML (120) and activates the STAT signal pathway in NSG mice after transplantation, which is consistent with the development of human primary diseases. Human UCB-Lin^-^ cells transfected with TEL-JAK2 engrafted into NOD-SCID mice showed that the graft tilted toward the myeloid and erythroid lineages and induced bone marrow fibrosis (123). Some co-expression methods further promote model construction and guide treatment, such as MLL-AF9 and NRAS co-transfection of human UCB-CD34^+^ cells induced faster AML progression in NSG mice (121); BCR-ABL and BMI1 co-transfected with human UCB-CD34^+^ cells induced B-ALL in NOD-SCID mice confirmed that BMI1 was a potential therapeutic target for CML (118);Human CD34^+^ cells transfected by DEK-NUP214 induced AML in NSG mice, demonstrating that the HOX family and the primary human t (6; 9) AML were highly up-regulated and became a potential target for treatment (119); Unlike conventional Notch mutation induced T-ALL mouse model (126), overexpression of Notch1 in human HSCs only resulted in alteration of human T cell differentiation tendency in NOG mice, while only 1/26 transplanted mice were found with T-ALL development (127) (**Table 1**, **Figure 3**).

**Table 1:** Mouse models with spontaneous human hematological malignancy development.

Disease		Gene editing subtype	Method	Reference	Characteristics of forming disease
Leukemia	B-ALL	MLL-AF9	Retroviral transduction	(114, 115)	MLL-AF9 induced pre-B-ALL; MLL-ENL induced B-ALL; High penetrance and short latency with a median of 7 weeks and lineage switching similar to MLL-rearranged patients at relapse.
		MLL-ENL	Retroviral transduction	(115)	
	CLL	Cancer associated-POT1 mutation	Crispr-Cas9 engineered	(116)	Resolution of the complexities of how changes in telomere length impact cancer progression in the proper genetic context and ideally *in vivo* that recapitulates the telomere length and proliferation dynamics of CLL.
	CML	BCR-ABL1	Retroviral transduction	(117)	A block at the pre-B-cell stage similar to CML patients.
		BMI1 + BCR-ABL	Retroviral transduction	(118)	With transformation biased toward a lymphoid blast crisis.
	AML	DEK-NUP214	Retroviral transduction	(119)	Recapitulation of primary human t(6;9) AML within an average of 6 months.
		ZMYM2-FGFR1	Retroviral transduction	(120)	Development of myeloproliferative disease that progresses to AML with a long (> 12 months) latency period; Successively transplanted through three generations.
		MLL-AF9+ NRAS;	Retroviral transduction	(121)	Significant and reproducible decrease in the latency of disease and quick development of AML.
		AML1-ETO+CBFβ-MYH11	Retroviral transduction	(121)	
T- and B-cell lymphomas		CNTRL-FGFR1	Retroviral transduction	(122)	Transdifferentiated into lymphoma during serial transplantation. Gene expression similar to CNTRL-FGFR1^+^ patients.
Myelofibrosis		TEL-JAK2	Lentiviral transduction	(123)	High levels of human engraftment at 3 weeks posttransplant; With decreased B lymphoid cells and increased myeloid cells; With rapidly progressive bone marrow fibrosis and anemia arising in the absence of concomitant splenomegaly with cell nonautonomous effects on the murine megakaryocytic lineage. With an increase in the level of STAT5 phosphorylation.

## 4 Optimization of human hematological malignancy mouse models

Other than immunological factors, non-immunological factors may also constrain the recapitulation of human malignancy in mice. Many efforts were input to optimize human hematological malignancy mouse models from this aspect in the last two decades (128). The microenvironment of hematological malignancies is composed of the bone marrow, blood vessels, and peripheral lymphoid organs providing nutrition and survival factors, which can affect the survival and growth of tumor cells. The Bone marrow microenvironment includes HSC and non-hematopoietic cells, and the latter includes endothelial cells, fibroblasts, osteoblasts, macrophages, mast cells and mesenchymal stem cells (MSCs). Because mouse bone marrow microenvironment is different from the human counterpart, it may not support the development of some less-invasive human hematological malignancies. The application of human cytokines and reconstruction of the bone marrow microenvironment further optimized the mouse model to reproduce the human microenvironment and address the limitations caused by differences between human and mouse microenvironments.

### 4.1 Application of human cytokine transgenic mice

The differences in amino acid sequence of cytokines/ligands between species lead to the difference in cross-reaction between humans and mice, which may affect the survival and development of human hematopoietic cells in mice. Human cytokine transgenic mice can promote hematopoietic differentiation of human myeloid cells and other cells in mice, improve the development of human hematopoietic immune system in mouse models, and increase the implantation rate of human hematological malignant cells (**Table 2**).

**Table 2:** Human cytokine transgenic mice for human hematological malignancy investigation.

Human cytokine	Method	Mouse strain	Efficacy	Indication
SCF, GM-CSF and IL-3	Knock in	NSG-SGM3, conducted on NSG	Improvement in the expansion of normal mature human myeloid cells; increased production of myeloid cells and T-reg	Benefit engraftment of AML, MDS, CLL (129, 130);
SCF	Transgene	Conducted on NSG	Improvement in irradiated NSG HSC engraftment and elimination of radiation-related complications (131)	
SCF/KIT ligand	Transgene	Conducted on NSG	Improved human granulocyte lineage and development of human myeloid lineage (132)	
IL-15	Knock in	Conducted on Rag2-/-*IL2rg*-/-	Increased proportion and functional maturation of human NK and CD8^+^ T cells and improved antibody-dependent cell-mediated cytotoxicity (ADCC) of anti-CD20 antibody (133)	Benefit engraftment of Burkitt lymphoma, enable NK cell-targeted immunotherapy of tumor xenografts
IL-6	Knock in	Conducted on NOG	Facilitation on studies of myeloid-derived suppressor cells in MM (134)	Benefit engraftment of MM
M-CSF, IL-3, GM-CSF, TPO	Knock in	MISTRG conducted on Rag2-/-*IL2rg*-/-	Support of human bone marrow monocyte development and strong human innate immunity to infection, as well as the development of NK cells and CD163^+^ tumor-infiltrating macrophages (135)	Benefit engraftment of MM
M-CSF, IL-3, GM-CSF, TPO, IL-6	Knock in	MISTRG6 conducted on Rag2-/-*IL2rg*-/-	Improvement in engraftment of human malignant plasma cells with nonmalignant cells (136)	Benefit engraftment of MM
TPO	Knock in	conducted on Rag2-/-*IL2rg*-/-	Increase in myelomonocytic cells, CD66^+^ granulocytes in the bone marrow	Benefit engraftment for those with specific subtypes of leukemia such as t (8;21)-ETO AML (137, 138)

NSG-SGM3: AML and other myelopoietic hematological malignancies are difficult to transplant into mice, which may be due to the lack of hematopoietic microenvironment and cytokines supporting homing, expansion and stromal cell growth of myelopoietic hematological malignant cells in mice. NSG-SGM3 transgenic mice with human stem cell factor(SCF), granulocyte-macrophage colony stimulating factor(GM‐CSF), and interleukin (IL)‐3 (121) promote the research of human myelopoietic hematological malignancy mouse models by increasing the production of mature myeloid cells (139) and enhancing the implantation rate of AML (129),MDS (140), and CLL (141). In terms of AML, some with difficulty in migration include those with AML1-ETO and CBFβ-MYH11 AML-LSC cells that can be successfully implanted in NSG-SGM3 mice. After implantation, the level of human CD45^+^/CD33^+^myeloid cells were significantly higher than that of NSG, and the transplanted cells expand with time, providing research opportunities for diseases that are difficult to model in mice.

MISTRG: Rongvaux et al (142) knocked in human cytokines macrophage colony stimulating factor (M-CSF), IL-3, granulocyte-macrophage colony stimulating factor (GM-CSF), and thrombopoietin (TPO) into RAG-/-*IL2rg*-/- mice, replacing the corresponding cytokine epitopes of mice, and human *SIRPA* genes were knocked in at the same time to produce MISTRG mice. The lack of corresponding cytokines in mice reduces the affinity of the niche in the bone marrow to mouse HSPCs, which reasonably provides additional benefits for human hematopoiesis. MISTRG mice support the development of human bone marrow mononuclear cells, NK cells and CD163^+^ tumor infiltrating macrophages. MDS stem cells cannot produce colony forming units *in vitro*, and their proliferation and differentiation abilities are defective. MISTRG mice can implant hematological malignant cells of different risk levels, including MDS, and maintain the genetic complexity of the original samples of hematological malignancies. MISTRG mice can be transplanted twice and are superior to NSG mice in terms of implantation rate and construction percentage of myeloid system (135). For MISTRG mice, the MDS implantation rate can reach 50%, which is significantly better than 10% of NSG. They can reproduce erythrocyte and megakaryocyte dysplasia in MDS, forming circular fibroblasts and dysplastic megakaryocytes with reticulin fibrosis.

IL-6-tg: IL-6 is the key growth factor of human MM, and human and mouse IL-6 lack cross-reactivity. Human IL-6 transgenic mice (136) improved the implantation rate of MM, provided the niche key factor for MM growth in mice, and allowed the implantation of precancerous, malignant plasma cells and non-malignant cells, promoting the study of myoid-derived suppressor cells in MM immune mechanism.

TPO-tg: TPO has been proven to be a key cytokine supporting HSC maintenance and self-renewal and can promote the development of megakaryocytes and platelets. The development of bone marrow mononuclear cells in immunodeficient mice, such as NSG, is relatively small, and the implantation level usually begins to decline 4-6 months after transplantation, which may be due to the difference of TPO between humans and mice. Human TPO transgenic mice promoted the development of human bone marrow mononuclear cells (137) and CD66^+^ granulocytes, and may improve the transplantation rate of leukemia with specific subtypes such as t(8; 21)-ETO AML (138).

### 4.2 Bone marrow microenvironment optimization and reconstruction

In addition to human hematopoietic system cytokines, other complex factors (such as ligands, chemokines, and hormones) in the bone marrow microenvironment also significantly affect the occurrence of hematological malignancies. For example, the exogenous cytokine c-kit ligand can improve the implantation of normal and leukemia CP-CML (chronic phase of chronic myeloid leukemia) cells (143); SCF tg improves the implantation of irradiated NSG HSCs and eliminates radiation-related complications (131, 132),SCF/KIT ligand tg improves the development of human granulocyte lineage and human myeloid lineage (132). Therefore, remolding the mouse bone marrow microenvironment or reconstructing artificial bone marrow tissue is another important way to optimize the mouse model of human hematological malignancy.

### 4.3 Combined injection of MSC

MSC creates a favorable environment for human MDS-derived cells to survive in the mouse microenvironment, possibly through the physical interaction itself and the production of human cytokines (144). Co-injection of patient hematological malignant cells with MSCs can promote the successful construction of PDX mouse model (66), and successfully build a hematological malignancy model that is difficult to implant, such as MDS. The majority of MSCs used are collected from healthy people, while some MSCs were established from patients’ CD34^−^ cell fraction (66). Injecting genetically modified MSCs platelet-derived growth factor subunit B, human IL-3 and TPO, into the tibia can further promote the survival, renewal and reduction of clone drift of HSCs (145), including the secondary transplantation (146). However, the presence of non-bone marrow-derived stromal cells (dermal fibroblasts) or non-stroma cells is not conducive to the implantation of MDS-derived cells. Also, for leukemia, co-injection of MSC and AML cells improve engraft rate, helping to evaluate key factors in the progression of leukemia in bone marrow niche (147, 148).

### 4.4 Reconstruction of artificial bone marrow microenvironment

Humanized bone marrow-like structures, including 3D scaffold, ossicles, and rabbit bone, can not only create artificial bone marrow microenvironment to better implant hematological malignant cells but also be used to screen for drugs, especially those targeting matrix components.

The “three-dimensional (3D) scaffold” coated with MSCs can be adjusted to form humanized bone tissue, which can also provide niche for implantation and homing of human UCB-CD34^+^ HSCs and the growth of cells in patients with hematological malignancies *in vivo* (149). The scaffold model retains the original clone structure. The flexible nature of the biocompatible cell carrier helps achieve the required size by simple cutting. Simultaneously, it also allows the use of collagenase for effective digestion, facilitating access to cells for further research. Usually, MSCs expanded *in vitro* are inoculated in gelatin sponge and cultured for several days, and then human hematological malignant cells are injected into the sponge, and then subcutaneously injected into non-irradiated immunodeficient mice (149), which is an effective *in vivo* model for studying the human hematopoiesis niche (150). In one study, MDS was only observed in mice co-injected with patient-derived MSCs and CD34^+^ cell bone (151), of which the regeneration rate of the method based on 3D bone was the highest (152);

“ Ossicles “ refers that bone marrow-MSC mixed with matrigel and subcutaneously injected into immunodeficient mice. After 2-3 months, they form “ossicle body”, which forms the external bone structure around the hematopoietic core (153), which also provide site for malignant and normal HSC to home and improve their engraftment and differentiation. Pievani e tal (154) created leukemia microenvironment using patient-derived MSCs *in vivo* and found MSCs improve the leukemia engraftment; Implantation of cartilage pellets followed by MSCs subcutaneous transplantation into NSG mice, bone marrow-stromal progenitors from AML mice have an increased adipogenic differentiation ability (155). The implantation of primary AML cells can be successfully achieved by transplanting subcutaneously injected polyurethane scaffolds, ceramic scaffolds or matrix gel coated with freshly separated human bone marrow-derived MSCs *in vivo (* 156), which proves that tumor cells or transplanted AML cells rich in CD34^+^ can circulate between ossicles.

For MM, there are already advanced SCID mice models with artificial humanized bone marrow microenvironment such as SCID-hu (human fetal bone) (157),SCID-rab (rabbit bone) (158), SCID-synth-hu (the 3D polymeric scaffold previously composed with human bone marrow stromal cells) (159). SCID-rab uses rabbit bone (158) to avoid the ethical problem of human fetal bone caused by SCID-hu and uses rabbit bone subcutaneously implanted in mice. Immunohistochemical analysis shows that most bone marrow microenvironment cells originate from rabbits. Direct injection of MCs from 28 patients into the implanted bone can successfully implant tumor cells from 85% of patients with MM and lead to the production of patients’ M protein isoforms and typical myeloma manifestations. MCs only grow in rabbit bones, but can transferred to another bone in the same mouse at a distance; Cells from patients with extramedullary diseases also grow along the outer surface of the rabbit bone. The system can now be widely used to study the biology and expression of myeloma and to develop new treatment methods for the disease.

Others: Denervation of bone marrow in recipient mice by using 6-hydroxydopamine can also improve the implantation rate of AML by changing the bone marrow microenvironment (160); NHL tumor microenvironment (161) is maintained by implanting BCL2 ^+^ and CD20^+^ lymphocytes (48).

## 5 Humanized mouse models with human immune cell composition and human hematological malignancy development for cancer immunotherapy study

Immunotherapy is a powerful tool to treat a variety of human hematological malignancy clinically. Immunotherapy for hematological malignancies includes non-myeloablative transplantation, T-cell immunotherapy, NK cells, immune-agonists, monoclonal antibodies (mAb) and vaccination (1). The history of immunotherapy for hematological malignancies began with mAb therapy in B-cell lymphoma (162), and then transited to gene-modified T cell therapy. These T cells were designed to recognize and target specific antigens, such as anti-CD19 Chimeric Antigen Receptor (CAR)-T cell therapy, and produced significant results in patients with B-cell malignant tumors (163). The advantage of immunotherapy is that it reproduces the microenvironment of immune cells and improves the overall survival rate of patients. The Food and Drug Administration (FDA) has studied and approved several immunotherapy programs for hematological malignancies, but many programs are still in the late stage of clinical development, including adoptive cell transfer, antibody-based therapies, and immune checkpoint inhibitors (1). In addition, patients with hematological malignancies who receive immunotherapy, especially CAR-T therapy, will also relapse (164), and have toxic reactions (165). The mechanisms include memory response and the source of immune cytokines, and many patients cannot benefit from current immunotherapy. To sum up, to minimize the risk of clinical trials, better animal models *in vivo* are needed for further research (166) to develop new immunotherapy schemes and prolong the survival period of patients. The early human hematological malignancy mouse model of immunotherapy was directly infused with corresponding immunotherapy cells, such as CAR-T, on the basis of CDX/PDX to verify the curative effect (167). However, they rely on the immune system of mice and cannot represent the real human immune therapy response. Therefore, there is an urgent need for a mouse model of the human functional immune system to reproduce the immune surveillance/immune regulation of humans to further study the complex response of immunotherapy in the human body.

The mouse model with human functional immune system focuses on the study of human immune surveillance, immune regulation (84) and pro-inflammatory cytokines, which can verify the immunotherapy of various hematological malignancies, including immune-cytokine therapy (168,)bispecific antibody (169), mAb (170), and CAR-T therapy (171). At present, the humanized mouse models applied to hematological malignancy mainly include Hu-PBL (human PBMC), Hu-SRC (SCID repopulating cell) and Thy/HSC models (also known as BLT models) (10).

Hu-PBL model was a humanized PDX/CDX model constructed by transplanting human PBMCs, which were easy to process and obtain, and by inoculating hematological malignant cells. In this model, human lymphocytes were rapidly expanded in mice, which can be used to study the interaction between human immune cells and hematological malignancies (172). However, because the injected cells are human mature immune cells, human myeloid cells and B lymphocytes are rarely detected in this model. Activated human CD4^+^or CD8^+^ T cells in mice often leads to severe graft-versus-host disease (GVHD), which markedly limits the observation period and making its wide application difficult.

Another popular humanized mouse (173) model is Hu-SRC model. It was constructed by transplanting human cord blood-, fetal liver-, bone marrow-or G-CSF-mobilized peripheral blood-derived CD34^+^ cells into irradiated neonatal immunodeficient mice. This model has sustainable multilineage human immune cell (including T cells, B cells and myeloid cells) reconstitution. This model can reproduce the primary immune reaction of T cells in humans. Among them, human T cells develop in the mouse thymus, and after positive and negative selection, this model has mouse MHC restriction (174). However, residual innate immunity, poor human thymopoiesis, immature B cells, lack of human leukocyte antigen (HLA) restrictions, and poor T cell-dependent humoral responses also exist.

Thy/HSC model (also known as BLT model) (90) is generated by co-transplantation of human fetal liver CD34^+^ cells (intravenous injection) and thymus tissue (under renal capsule) into sublethal-total-body-irradiated immunodeficient mice. This model has high levels of human immune system reconstruction and secondary lymphoid organ well development. HSCs extracted from human liver tissue have more efficient transgenic expression. The Thy/HSC model can generate specific T cell and antibody responses against protein antigens, virus antigens, autoantigens, allogeneic and xenogeneic cell antigens, which proves that it can generate strong human immune responses. Transplanted human liver and thymus provide a microenvironment that supports the development of human T cells. Hassall’s corpuscles formed by human stromal cells and T progenitor cells can be detected, which can produce a human HLA-limited human T cell immune response. Mouse DCs can migrate to the human thymus and induce human T progenitor cells, reducing xenograft rejection.

Application of humanized mice in the immunotherapy of hematological malignancies: mainly embodied in the verification and exploration of immune checkpoint inhibitors, cytokine therapy, and cell therapy, such as CAR-T. (**Table 3**)

**Table 3:** Part of Investigational New Drug (IND)-approved drugs that utilize mouse models with human hematological malignancy for pre-clinical tests between 2020-2023.

Hematological malignancy	Current IND-approved drug name	Model	Reference
AML	Entrectinib (Trk inhibitor)	CDX	(175)
ATL	anti-CC chemokine receptor4 (CCR4) CCR4 mAb	PDX with autologous human immune cells	(176)
MM	Teclistamab	CDX/PDX inoculated with human T cells (hu-PBL)	(177)
AML	AG-120 (ivosidenib)	xenograft mouse model	(178)
ALL	CD19 CAR-T cells	PDX	(179)
Leukemia	Anti-CD52 mAb	CDX	(180)
T-ALL, B-ALL	Isatuximab (CD38mAb)	Xenograft models	(181)
Lymphoma (NHL)	HX-009 (bispecific antibody, targeting PD-1 and CD47 but with weakened CD47 binding)	CDX	(182)
Lymphoma	PD-1 antibody combined with CTLA-4 antibody	EBV-induced model (virus induced model)	(6)
DLBCL	AZD4573 (a selective inhibitor of cyclin-dependent kinases9)	CDX	(183)
AML DLBCL	HexaBody-CD38	PDX	(184)
relapsed/refractory MM	ISB 1342 (a CD38 × CD3 T-cell engager)	CDX injected with human PBMC (hu-PBL)	(185)
ALL	Radiotherapy combined with CD19-CAR-T cells	CDX	(186)
ALL	Duvelisib	PDX	(187)
ALL AML MM	LAVA-051	CDX or CDX with human PBMC (hu-PBL)	(188)
MM	elotuzumab	PDX (SCID-hu)	(189)
MM	daratumumab plus All-trans-retinoic acid (ATRA)	CDX with humanized microenvironment (scaffold)	(190)
NHL	Tazemetostat (EPZ-6438)	CDX	(191)
NHL	AZD0466	CDX	(192)
MM	CD38 CAR-T cells	CDX with humanized microenvironment (scaffold)	(193)
Leukemia	CD-7 CAR-T cells	CDX	(194)

Immune checkpoint inhibitors: The application of immune checkpoint inhibitors in hematological malignancies mainly includes CD137 antibody, programmed cell death protein-1 (PD-1) antibody, or cytolytic T lymphocyte-associated antigen (CTLA)-4 antibody, and their effect can be verified in humanized mouse models, with either single therapy or combined therapy for different types of tumors. The efficacy of anti-CD137 antibody was tested in MM CDX humanized mice models with inoculation of human healthy NK cells (hu-PBL) (195). The effect of PD-1 and CTLA-4 antibodies were evaluated in EBV induced B cell lymphoma humanized mouse models (196) before clinical trial. The human immune cells of humanized mice can mediate the immune response against therapeutic antibodies. For example, the effectiveness of bi-specific CD20/CD3 antibody can also be verified in a lymphoma CDX hu-PBL humanized mice model (169). However, these humanized mouse models have different immune cells from human donors, which cannot completely replicate the immune response in patients with tumors.

For cytokine therapy, humanized mice with cytokines can reproduce the immune response in patients during immunotherapy. For example, human IL-2 transgenic mice (197) produce various NK cells, which can reproduce human NK cells targeted to implanted leukemia and lymphoma cells (198);Human IL-15 knock-in (199, 200) humanized mice better mimic human NK (201)and CD8^+^T cells that remain in human circulation and tissue to target Burkitt lymphoma, as well as ADCC effect on CD20 antibody (68, 133).

For cell therapy, humanized mice can represent its immune effect and toxicity in humans *in vivo*. Recently Caruso et al. described a humanized mice model system constructed with human HSCs, AML cells and human endothelial tissues to evaluate the on target-off tumor toxicity of CAR.CD123-NK cells. This is a new model for studying the off-tumor toxicity of immunotherapy for hematological malignancy, especially for relapsed/refractory pediatric AML (202). Additionally, humanized mice can reproduce cytokine release syndrome (CRS) and neurotoxicity in patients after infusion of CAR-T cells. For example, Mhaidly et al (203) experimented in NSG mice with CD19^+^human B-lymphoblastic leukemia, delivering anti-CD19 CAR by CD8 targeting lentivirus and injecting human PBMC (204). Compared to the control, a single injection was sufficient to eliminate tumor cells. However, its CAR-T cells came from allogeneic and HLA donors (205). Although the occurrence of CRS was the same as that of CAR-T patients (206), CRS could not be distinguished from allogeneic reactions. Therefore, it is necessary to verify the humanized spontaneous model of human hematological malignancy by monitoring auto-functional human immunity.

The humanized mouse model with homologous immune system and spontaneous human leukemia development is a novel mouse model recently developed by our group for human hematological malignancy investigation. It is mainly based on the Thy/Liv SCID-Hu mouse model (207). The model was constructed by transplanting CD34^+^cells from human fetal liver and human fetal thymus from the same embryo donor into NSG mice with sublethal radiation. CD34^+^ cells were transduced by a retrovirus carrying the MLL-AF9 fusion gene and GFP. About 10-12 weeks after humanization, human immune cells were reconstructed, human CD3^+^ T cells from spleen were isolated, transferred with a virus vector encoding human CD19 specific CAR, expanded *in vitro* for about 2 weeks, and then returned to leukemic humanized mice with adoptive immunotherapy (208, 209). In most previous studies, the reconstructed human immune system and inoculated human tumors are allogeneic. The allogeneic response of human T cells to malignant tumors may damage the value of data collected from these models, making it difficult to predict the clinical immune effects of anticancer drugs (10). While this model allows the evaluation of anti-tumor response in “immune-active” hosts without allogeneic or xenogeneic immune response. CAR-T cells are modified autologous T cells developing in human thymus. In mouse blood, we can detect anti-CD19 CAR-T cells, whose dynamics and levels are similar to those of patients receiving CAR-T, and are closely related to the burden of leukemia, B-cell destruction and mouse survival, which is helpful to identify new and more effective CARs (210), and inhibit side effects, such as CRS and neurotoxicity. In this model, human immune cells and leukemia cells were derived from CD34^+^ HSCs from the same human fetal liver. These cells are considered autologous and have a homologous immune monitoring system. Even the CAR-T produced by humanized mice will not attack mice, nor will it cause strong xenograft rejection. The humanized mouse model of B-ALL induced by MLL-AF9 can also verify the treatment of receptor leukocyte infusion (RLI) without GVHD (211),which has a similar immune response to patients. For instance, increase in the proportion of cytokines produced by T cells and mononuclear macrophages and Treg reveals that GM-CSF may play a key role in CRS after CAR-T cell treatment; and RLI can improve the anti-tumor efficiency by depletion of human T cells (211). In addition, Leskov et al. developed a “double-hit” lymphoma model and constructed a humanized mouse model of lymphoma with the human immune system by inducing joint overexpression of c-MYC and BCL2 in human HSC-derived B-lineage cells through lentivirus transduction. This model accurately summarizes the histopathological and clinical features of human “double-hit” lymphoma resistant to steroids, chemotherapy and rituximab (212), and can evaluate immunotherapy.

## 6 Discussion and summary

Advancement in mouse models with human hematological malignancy recapitulation intensively contributes to exploring the pathological mechanism of related human diseases and facilitates invention of effective drugs and therapeutic approaches in last two decades. Development of mouse strains with severe deficiency of murine immune system (such as NSG mice) almost completely prevent mouse versus human immunological rejection after human cell/tissue transplantation and markedly elevate the successful rates of CDX and PDX model construction. Whereas non-immunological factors still constrain the capability of immunodeficient mice to accept and repopulate less aggressive human hematological malignant cells, especially the ones closely rely on human specific microenvironment to survive and differentiate. Thus, development of immunodeficient mouse strains with delicate human cytokine/chemokine/ligand expression or reconstruction of artificial human bone marrow microenvironment would further promote their application in human blood disease study. Importantly, proper combination of humanized mouse model with functional human immune system and the mouse model with human hematological malignancy development may further generate a powerful tool to precisely evaluate the efficacy of new drugs or immunotherapies and speed up their translational paces in clinic.

## Author contributions

YL and YNL performed literature search and wrote the manuscript. YT and ZH conceived the framework of this article, wrote, and edited the manuscript. All authors contributed to the article and approved the submitted version.
